# Increased Brain‐to‐Brain Synchronization During Literary Arabic Storytelling Following a Dialogic Reading Intervention: A Hyperscanning‐EEG Study

**DOI:** 10.1002/brb3.71003

**Published:** 2025-11-21

**Authors:** Georgina Abu Ghanima, Merna Haj, Azhar Badarne, Rola Farah, Areej Masarwa, Tzipi Horowitz‐Kraus

**Affiliations:** ^1^ Educational Neuroimaging Group, Faculty of Education in Science and Technology Technion‐ Israel Institute of Technology Haifa Israel; ^2^ Faculty of Biomedical Engineering Technion‐ Israel Institute of Technology Haifa Israel; ^3^ TecHMRC, Technion Human MRI Research Center Technion‐Israel Institute of Technology Haifa Israel; ^4^ Al‐Qasemi Academic College Hafia Israel; ^5^ Maktabat Al‐Fanoos Ramat‐Gan Israel; ^6^ Department of Neuropsychology, Center for Neurodevelopmental and Imaging Research (CNIR) Kennedy Krieger Institute Baltimore Maryland USA

**Keywords:** brain‐synchronization, hyperscanning, intervention, literary Arabic, parent‐child reading

## Abstract

**Purpose**: Arabic is unique in having two forms—literary Arabic (LA) and spoken Arabic (SA)—which present challenges in maintaining children's attention during the reading acquisition stage. Dialogic reading (DR) is an intervention that encourages children to actively engage in storytelling, thereby fostering attention, early language development, literacy, and executive function skills. Methods: This study examined the impact of DR in LA on joint attention during storytelling among 13 preschoolers (aged 48–60 months) and their mothers, using a hyperscanning electroencephalogram (EEG) method. Behavioral and inhibition tasks were also employed to assess the intervention's effects. Finding: Paired t‐tests revealed a smaller difference between correlation coefficient matrices for LA versus SA synchronization after the intervention compared to before. Behaviorally, significant improvements were observed in listening comprehension, executive functions, and processing speed following DR. Furthermore, enhanced inhibition was associated with a smaller difference between SA and LA synchronization following the intervention. Conclusion: These findings suggest that DR not only promotes language development but also reduces the neural disparity between SA and LA, highlighting its potential to support early cognitive and linguistic development in Arabic‐speaking children.

## Graphical abstract

A smaller difference between parent‐child brain synchronization for Literary Arabic (LA) versus Spoken Arabic (SA) after the intervention, compared to before. Following the DR intervention, greater scores in listening comprehension, executive functions, and processing speed tests were found. Enhanced inhibition was associated with a smaller difference between SA and LA synchronization following the intervention.

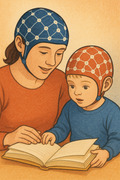

## 1 Introduction

### 1.1 The Unique Nature of Arabic: Diglossia and Its Implications for Reading Development

Arabic is a semitic language and one of the most widely spoken languages in the world (Arabic | 34 | v3 | The World's Major Languages | Alan S. Kaye | Taylor 2018). It is written from right to left, consisting of 28 letters that function as root radicals. Like other Semitic languages, Arabic is characterized by a non‐concatenative morphological structure (Saiegh‐Haddad and Henkin‐Roitfarb 2014). Spoken Arabic (Ammiyya) is acquired in early childhood and used as an informal language in daily communication (Saiegh‐Haddad and Henkin‐Roitfarb 2014; Ibrahim 2011). Different Arab countries have distinct varieties of “Ammiyya” (spoken Arabic), and within each country, multiple dialects may coexist. In contrast, literary Arabic (Fusḥa), which individuals are first exposed to in school, is considered the formal and standardized language (Saiegh‐Haddad and Henkin‐Roitfarb 2014; Ibrahim 2011). This linguistic phenomenon, known as diglossia, refers to the coexistence of two language forms, spoken and literary, that differ significantly in grammar and phonology. Diglossia has been linked to lower educational outcomes, including difficulties in reading and writing (Diglossia in Arabic: Investigating Solutions; The Effect of Diglossia on Literacy in Arabic and Other Languages). These challenges can negatively impact students' academic performance and adult literacy levels. Beyond these educational outcomes, diglossia also imposes additional cognitive demands, as processing literary Arabic (LA) requires greater attentional and control resources compared to the more familiar spoken Arabic (SA) (Arabic | 34 | v3 | The World's Major Languages | Alan S. Kaye | Taylor 2018; Saiegh‐Haddad and Henkin‐Roitfarb 2014; Ibrahim 2011; Johnson 2011). To mitigate these effects, parents can support their preschool children by enriching their vocabulary and promoting early literacy through shared reading experiences. One effective approach to achieve this is called dialogic reading (DR).

### 1.2 Dialogic Reading as a Method for Triggering Executive Functions and Joint Attention in a Story

Dialogic Reading (DR) is an interactive reading method backed by evidence. It helps improve children's education and cognitive growth (Howard et al. 2016). Whitehurst and his colleagues originally developed DR to engage the child in storytelling. In this approach, the adult does not just read aloud; instead, the child becomes actively involved (Whitehurst et al. 1998; Whitehurst et al. 1994). In contrast to regular shared reading, where the adult reads while the child listens with minimal input, DR uses a structured role‐reversal. Here, the child gradually takes on the role of the storyteller, with the adult providing guided support (Whitehurst et al. 1994; Bus et al. 1995). This support follows the PEER sequence (prompt, evaluate, expand, repeat) and uses CROWD prompts (completion, recall, open‐ended questions, wh‐questions, distancing). These strategies help adults engage children in meaningful conversations about the text (Whitehurst et al. 1994; Flynn 2011). During this process, the adult asks wh‐questions and open‐ended questions. They follow up on the child's answers by expanding, repeating, and elaborating on them while also praising and encouraging participation (Flynn 2011; Parish‐Morris 2013). These techniques not only boost early literacy but also help develop vocabulary, listening skills, attention, and executive function (Howard et al. 2016).

Executive functions (EF) are mental processes needed for focusing and paying attention, these functions are necessary for mental and physical health, cognitive, social, and psychological development (Diamond 2013). More specifically, these abilities include working memory, which assesses the ability to hold information in mind and mentally work with it (Miyake and Friedman 2012). For instance, reading aloud letters, recalling them in sequence, deleting the first letter then adding a new one (Miyake and Friedman 2012), and shifting forwards and backwards between multiple tasks or mental sets (Miyake and Friedman 2012). Inhibition refers to the capability to control a child's behavior, thoughts, or emotions, allowing him to focus on what he chooses and restrain attention to other stimuli (Miyake and Friedman 2012).

The acquisition of literacy in Arabic places unique demands on EF because of the diglossic nature of the language. Children are required to navigate between two linguistic systems: SA and LA (Arabic | 34 | v3 | The World's Major Languages | Alan S. Kaye | Taylor 2018; Saiegh‐Haddad and Henkin‐Roitfarb 2014). This duality engages working memory, which supports the ability to hold and manipulate LA forms while comparing them to the more familiar SA, and inhibition, which enables the suppression of the dominant SA response during LA processing (Saiegh‐Haddad and Henkin‐Roitfarb 2014; Ibrahim 2011). Consequently, EF are central to the development of reading in Arabic, which provides a strong rationale for the expectation that dialogic reading (DR) training may enhance EF and will facilitate more efficient management of switching between LA/SA (Howard et al. 2016; Diamond 2013; Miyake and Friedman 2012). Emerging evidence indicated that processing LA, compared to SA, recruits additional neural pathways, reflecting the need for higher cognitive control and linguistic monitoring (Saiegh‐Haddad and Henkin‐Roitfarb 2014; Ibrahim 2011; Johnson 2011). It was found that a DR intervention in 4‐6‐year‐old children in Hebrew had a significant positive effect on EF and language abilities. The DR group children got greater visual attention and switching and had shorter reaction times. Neuroimaging studies provide converging evidence that DR enhances activation in brain regions supporting EF and attention, linking behavioral gains to neural efficiency (Johnson 2011; Hutton et al. 2017; Twait et al. 2019). These findings suggest that DR may not only improve EF outcomes but also strengthen the neural basis for coordinating parent–child interactions, laying the groundwork for enhanced brain‐to‐brain synchronization during shared LA reading.

Children undergo significant transformations in attentional control during their early development (Hutton et al. 2017). Studies have found that adult‐child storytelling influences the development of EF brain networks in preschoolers (Twait et al. 2019). Additionally, the interaction level between the mother and her child correlates with specific brain regions related to language, socioemotional, and EF in preschoolers (Howard et al. 2016). According to Posner and Petersen's study, those cognitive abilities comprise two systems (Petersen and Posner 2012). The first system is attentional control, primarily responding to external stimuli through bottom‐up processes (Petersen and Posner 2012). The second system is related to EF, a top‐down process that includes working memory, speed of processing, and error monitoring (Petersen and Posner 2012). These systems can be measured using functional MRI, focusing on specific networks (Miyake and Friedman 2012), using electroencephalogram (EEG), analyzing the data based on the event‐related potentials (ERP) (Twait et al. 2019). Another method of examining the allocation of attention to a given stimulus and characterizing processing (mainly when focusing on a joint activity) is using a hyperscanning methodology.

### 1.3 Hyperscanning as a Method for Measuring Joint Attention

Hyperscanning is a neuroimaging technique used to simultaneously record neural or circulatory brain activity in both the parent and child (Petersen and Posner 2012). When conducted using electroencephalography (EEG), hyperscanning offers the advantage of capturing brain activity at the millisecond scale, allowing researchers to examine the temporal dynamics of brain‐to‐brain connectivity between two individuals (Arabic | 34 | v3 | The World's Major Languages | Alan S. Kaye | Taylor 2018). Brain‐to‐brain synchronization within this framework refers to the temporal alignment of neural signals across partners. It can be quantified through measures such as phase locking, which captures the alignment of neural oscillations over time, and functional connectivity, which reflects correlated neural activity patterns that indicate inter‐brain communication (Lachaux et al. 1999).

Repeatedly, studies showed that during parent‐child interaction, a greater brain synchronization was observed compared to a less interactive condition (Arabic | 34 | v3 | The World's Major Languages | Alan S. Kaye | Taylor 2024; Zivan et al. 2022; Abu Elasal et al. 2020; Abdo et al. 2020). In particular, brain‐to‐brain synchrony between parent‐adolescent dyads using hyperscanning EEG while completing a picture processing task indicated that higher parental involvement was related to a better emotional state and communication patterns between the parent and their adolescent (Arabic | 34 | v3 | The World's Major Languages | Alan S. Kaye | Taylor 2024). Similarly, a reduced mother–child brain‐to‐brain synchrony was observed when storytelling is interrupted by media (smartphones) (Zivan et al. 2022), while other studies demonstrated a robust synchrony during storytelling (Abu Elasal et al. 2020), linking to a higher speech synchrony during dialogic reading with improved child cognitive skills (Abdo et al. 2020). These findings suggest that hyperscanning is a valuable method for capturing inter‐brain dynamics that reflect the quality of parent–child engagement. Importantly, brain‐to‐brain synchrony has been shown to align with behavioral measures of joint attention (Arabic | 34 | v3 | The World's Major Languages | Alan S. Kaye | Taylor 2024; Zivan et al. 2022; Abu Elasal et al. 2020; Abdo et al. 2020; Gashri et al. 2024). In diglossic contexts, where comprehension demands differ between LA and SA (Shahbari‐Kassem et al. 2024), synchrony may therefore serve as a neural marker of how effectively mother–child dyads manage attention to less familiar LA input.

Despite previously reported behavioral gains in executive function (EF) and language abilities among children exposed to DR, two key questions remain unresolved: (1) whether DR intervention in LA enhances EF and linguistic skills, and (2) whether DR in LA leads to increased brain‐to‐brain synchronization following the intervention. The present study aimed to address these gaps by using EEG to examine the effects of DR intervention in LA on EF and language performance and brain‐to‐brain synchronization in Arabic‐speaking children. We further hypothesize that, following the DR intervention, the disparity in brain‐to‐brain synchronization between LA and SA conditions will be reduced, and that this reduction will be associated with improvements in vocabulary skills.

## 2 Methods

### 2.1 Participants

Twenty‐five dyads participated in the current study (participants’ mean age: 5.15 years ± 0.647, 15 females; mothers’ mean age: 34.38 years ± 2.91). Of those 25 dyads, 15 children (mean age: 5.28 years ± 0.66, 7 females; mothers’ mean age: 34.86 years ± 3.14) completed the intervention and post‐intervention testing. The average age of maternal education was 12 years ± 1.30 with an average annual income of 18,362 ± 9070 NIS (1 dollar = ∼ 4 NIS). Inclusion criteria required participants to be between 4 and 6 years of age, enrolled in an Arabic‐speaking kindergarten, and native Arabic speakers. Additional criteria included the absence of psychiatric or neurological disorders and average to above‐average nonverbal cognitive abilities, as measured by the Matrix Reasoning subtest of the Wechsler Preschool and Primary Scale of Intelligence (WPPSI) (Wechsler 2002; Wechsler 2014). The study was approved by the institutional ethical committee. Participants were compensated for their time and travel with $50.

### 2.2 Study Procedure

**FIGURE 1:**
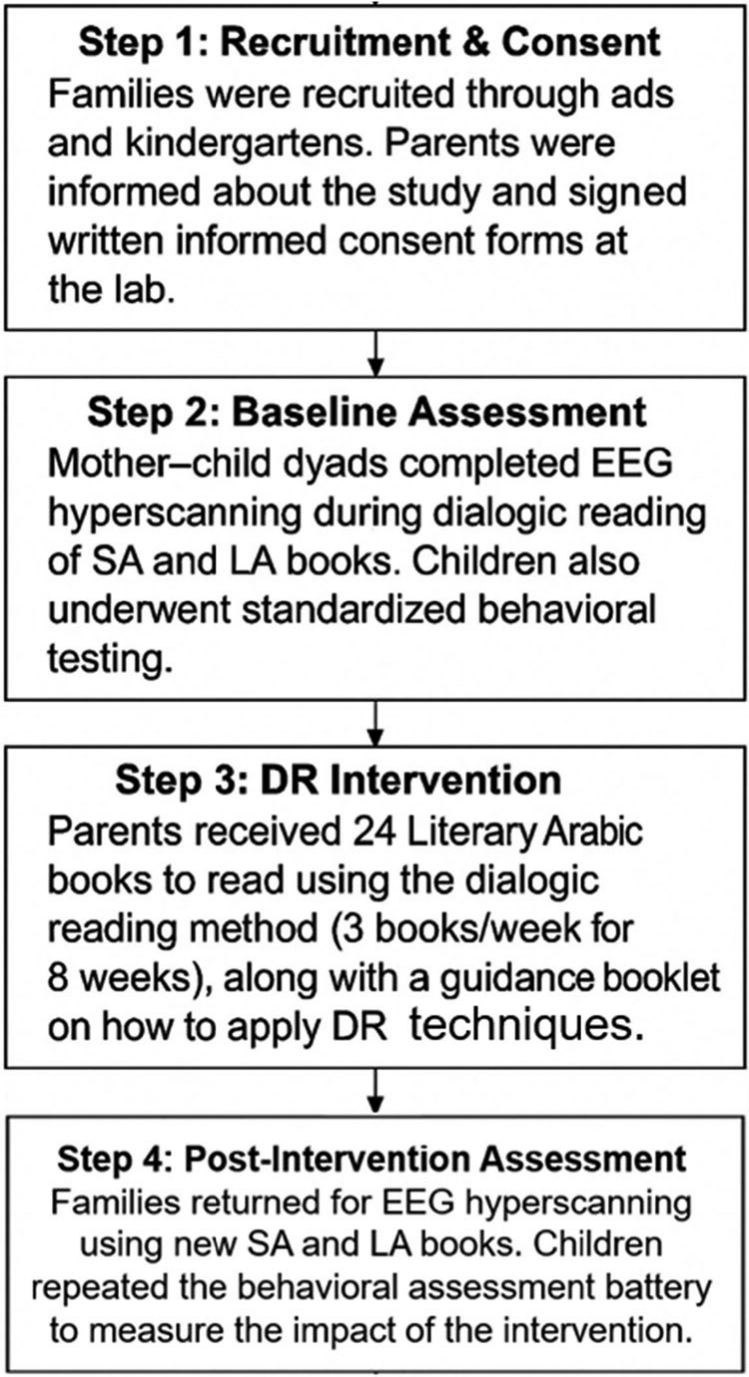
Flowchart describing the study procedure.

Families were recruited via posted ads in the laboratory's social media and via communications with local kindergartens in northern Israel. Participants were invited to the laboratory, and parents signed a written informed consent. Children then performed several cognitive (EF) and language tests, followed by hyperscanning‐EEG testing during two separate conditions: (1) SA reading and (2) LA reading (using two age‐appropriate books (see Supplemental material 1: examples for SA books), each having two versions– one in LA and another in SA, which were counterbalanced across participants). Then, children were given 24 age‐appropriate books for 24 DR sessions to be performed at home. The books included Literary Arabic wording (based on an educational expert's rating [Maktabat El Fanus]). Then, parents were provided with both written and verbal guidelines regarding the method of DR, including a booklet with an explanation outlining the importance of questions, vocabulary questions, pointing at the writing direction while telling the story, rhyming, and more (see supplemental material 2: Parents' Guide to Dialogic Reading). Parents were asked to record themselves during the home reading interventions and share the recordings with the study team for monitoring during the 8‐weeks intervention. After 24 sessions (3 times per week, for 8 weeks), the participants and their parents were invited to the lab for additional behavioral and EEG testing. In a similar design to Test 1, two books were used in the LA and SA versions also for the second EEG testing (Test 2), though with different books to avoid a priming effect. The list of books is in Supplemental Materials 2. See Figure 1 for the study's design.

### 2.3 Behavioral Measures

A range of standardized tests was administered to evaluate cognitive and language abilities. To assess executive function (EF) skills, the following tests were administered:

1. Working memory was assessed using the Digit Span subtest from the Comprehensive Test of Phonological Processing (Wagner et al. 1999).
2. Inhibition was evaluated in two modalities: in the visual domain, using the Walk/Don't Walk subtest from the Test of Everyday Attention for Children (TEA‐Ch) (Manly et al. 1999), and in the auditory domain, using the Score! subtest from the same battery.
3. Cognitive flexibility (switching) was assessed using the Animals/Colors test (Ziv 2017).

In addition to core EF skills, two cognitive abilities that support EF were also measured:

1. Visual attention, using the Sky Search subtest from the TEA‐Ch battery (Manly et al. 1999); and
2. Processing speed, using the Symbol Search subtest from the Wechsler Preschool and Primary Scale of Intelligence (WPPSI) (Wechsler 2002).

To assess language skills, participants completed vocabulary assessments from both the WPPSI (Wechsler 2002) and WISC (Wechsler 2014), assessing their SA and LA abilities, respectively. Phonemic awareness and naming abilities were assessed using the respective subtests from the Loghat Alkeraa linguistic Arabic‐based battery (Asadi et al. 2014).

### 2.4 Electrophysiological Data Acquisition

#### 2.4.1 EEG Task Conditions

EEG data were collected during two conditions: (1) book reading in SA and (2) book reading in LA. In both conditions, the mothers were asked to read the books to the child in their regular reading patterns. Each condition lasted approximately 5 min while EEG data were recorded. Immediately after reading each of the two books, the experimenter presented the child with four open‐ended narrative comprehension questions. Importantly, the LA and SA books were reviewed by an expert in Arabic child literacy to confirm the equivalence of the Literary Arabic (LA) and Spoken Arabic (SA) books.

#### 2.4.2 EEG Data Collection

EEG data were recorded using a 64‐channel EEG system (Brain Products, Germany). Each set consisted of 32 electrodes, including one electrode that was used as a reference electrode. One set was placed on the child's scalp, and the other on the mother's scalp, using the same electrode positions (10/20 system). The electrodes were connected to the amplifier, and each cap had its own ground electrode.

### 2.5 Data Analysis

#### 2.5.1 Behavioral Data Analysis

Several paired t‐tests (Test 1 vs. Test 2 data) were conducted to detect the effect of the DR intervention.

### 2.6 EEG Hyperscanning Data Analysis

#### 2.6.1 Preprocessing

EEG data were collected using a 64‐channel EEG system (Brain Products, Germany), which recorded the signal from the mother and the child using the BrainVision recorder application (Brain Products, Germany). For artifact removal, several steps were applied. Initially, the data were preprocessed using a band‐pass filter with 1 Hz and 45 Hz cutoff frequencies that removed high‐frequency noises and a notch filter at 50 Hz; an average reference was applied between maternal and child electrodes, which helped reduce common noises across the electrodes. Although applying filters improved the signals, eye movements, muscle activity, and cardiac signals still caused various artifacts. Therefore, additional artifacts were removed using the Independent Component Analysis (ICA) process (Winkler et al. 2011). Components were marked for rejection based on specific criteria: (1) Topography—eye components typically exhibited an anterior topography, while muscle components are usually localized at a single electrode; (2) Power spectrum—EEG signals typically showed a peak around alpha frequency (10–15 Hz), whereas eye‐movement components lacked this peak and muscle components exhibited higher power spectra as frequency rose; (3) Time course—eye movements often showed clear peaks corresponding to blinks; and (4) IC Label probabilities of above 0.6. Component rejection was conducted by two trained raters following these standardized criteria in combination with the ICLabel thresholding. Channels with artifact patterns and abnormal amplitudes were subsequently removed. This step eliminated non‐neural sources of noise, which enhanced the clarity of the EEG data.

#### 2.6.2 EEG‐Post Processing

After preprocessing, the continuous EEG data were segmented into 1000 ms epochs. Segmenting the data allowed for analyzing discrete, time‐locked neural responses and improved signal‐to‐noise ratio by averaging across similar time windows. This step is essential for aligning the EEG signals with specific events or time periods during the reading interaction. On average, 341 ± 90 valid epochs were retained per condition, and the mean signal‐to‐noise ratio was −1.56 ± 2.28 dB, indicating adequate data quality across conditions. Following the segmentation, a circular correlation matrix was created to quantify the level of neural synchronization between each electrode pair of the mother and her child. This method effectively analyzes EEG signals, which naturally display periodic and exhibit cyclical properties (Zivan et al. 2022). It measures synchronization by assessing the similarity between two cyclic signals across all phase shifts. This method is crucial for detecting correlations hidden by phase discrepancies, which conventional methods may miss. It enables the creation of a circular correlation matrix for parent‐child interactions, providing insights into complex neuronal activity patterns (Zivan et al. 2022).

Once the circular correlation process was completed, several correlation coefficient matrices were created: (1) Test 1, SA; (2) Test 1, LA, and the difference between the conditions; (3) Test 1 SA > Test 1 LA. To demonstrate the effect of the DR intervention, additional correlation coefficient matrices were created; (4) Test 2, SA; (5) Test 2, LA; (6) Test 2 SA < Test 2 LA; and (7) ([Test 2 SA > Test 2 LA] > [Test 1 SA > Test 1 LA]). For the EEG analyses, 961 electrode‐pair comparisons were tested. Results were corrected for multiple comparisons using a Bonferroni adjustment, yielding a corrected significance threshold of *p* < 0.000052.

### 2.7 Correlations Between Neuroimaging and Behavioral Measures

To determine the relations between the different correlation coefficient matrices and behavioral measures, Pearson correlations between the behavioral measures for the changes in LA versus SA vocabulary skills, EF abilities, and the EEG matrices for the following conditions (1) Test 1 SA > Test 1 LA; (2) Test 2 SA > Test 2 LA; and (3) ([Test 2 SA > Test 2 LA] > [Test 1 SA > Test 1 LA]) were conducted.

**FIGURE 2:**
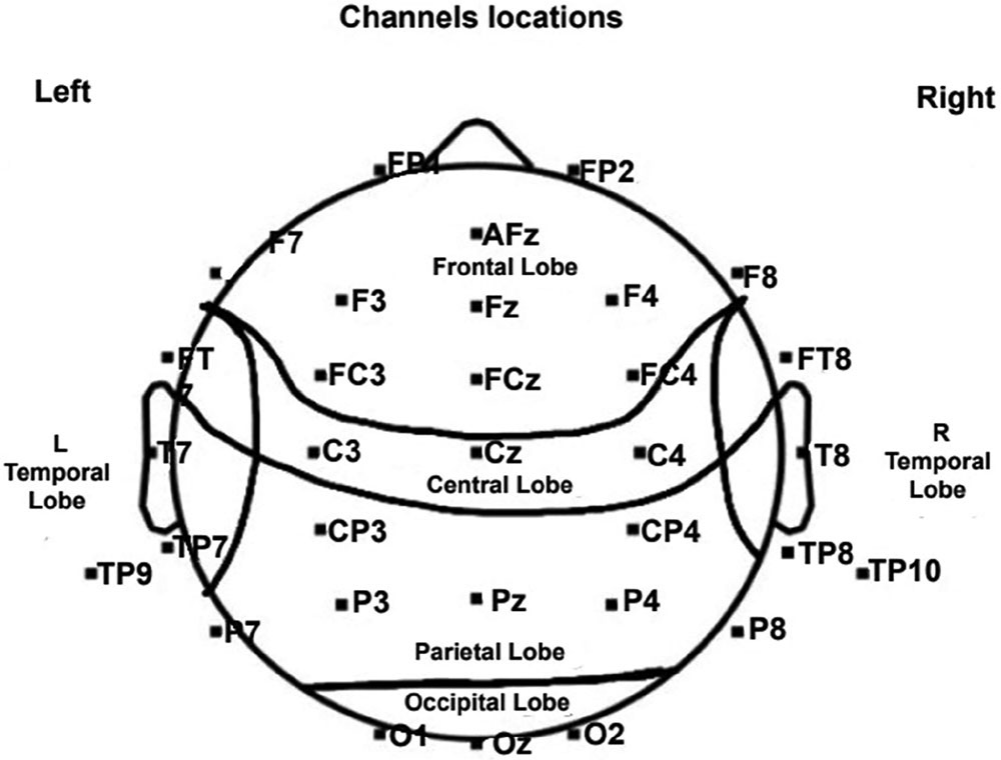
Electrode distribution per brain region.

To associate the changes following DR in mother‐child brain‐to‐brain synchronization with behavioral changes in EF and language abilities from Test 1 to Test 2, the electrodes were systematically organized per previous studies (Zivan et al. 2022) to ensure a precise brain topography mapping (see Figure 2). The correlations were performed only with brain‐to‐brain pairs that showed significant differences between the conditions.

In Figure 2, 32 electrode locations were used to record the brain region activity: right frontal (FP2, F8, F4, FC4, AFz), right central (C3, Cz), right temporal (FT8, T8, TP8, TP10), right parietal (CP4, P8, P4), left frontal (Fp1, F7, F3, Fz, FC3), left central (C3), left temporal (FT7, T7, TP7, TP9), left parietal (CP3, P7, P3), and the occipital region (O1, Oz, O2) electrodes.

## 3 Results

### 3.1 Behavioral Results

#### 3.1.1 Baseline Measures (Test 1)

Cognitive and language abilities before the intervention (Test 1) revealed results within the normal range and above for all participants (for the full sample; n = 25). See Table 1.

**TABLE 1:** Descriptive results for the cognitive and linguistic measures before intervention.

	Ability	Test	Mean	X (SD)	Min‐max	Normal range
General abilities	Nonverbal abilities	Matrix (WPPSI, standard score)	11.260	3.018	4.00–17.00	7–13
Language abilities	Language abilities	Vocabulary, LA (WPPSI, raw score)	14.550	4.914	6.00–27.00	—
		Vocabulary, SA (WISC, raw score)	18.238	6.647	9.00–35.00	—
	Narrative comprehension, SA	Comprehension (correct responses)	3.240	0.778	1.00–4.00	—
	Narrative comprehension, LA	Comprehension (correct responses)	3.708	0.464	3.00–4.00	—
	Naming	Naming objects time (Loghat Alkeraa, sec)	94.280	25.346	50.00–162.00	—
		Naming objects, accuracy (Loghat Alkeraa, number of errors)	0.800	0.912	0.00–3.00	—
		Naming letters, time (Loghat Alkeraa, sec)	163.266	63.990	54.00–237.00	—
		Naming letters, accuracy (Loghat Alkeraa, number of errors)	9.214	8.172	0.00–22.00	—
EF	Working memory	Digit span (CTOPP, standard score)	5.458	2.264	2.00–11.00	7–13
	Inhibition	Walk/don't walk, accuracy (TEA‐CH, number of correct responses)	2.727	1.120	1.00–5.00	—
		Score accuracy (TEA‐CH, number of correct responses)	4.434	1.618	1.00–6.00	—
	Switching	Switching (color, correct responses)	26.869	2.817	21.00–30.00	—
		Switching (animal, correct responses)	25.695	5.049	12.00–30.00	—
Supporting EF	Visual attention	Sky search, accuracy (TEA‐CH, age scale)	7.478	3.028	3.00–15.00	7–13
		Sky search, time (TEA‐CH, correct per second, age scale)	4.173	3.069	1.00–13.00	—
	Speed of processing	Coding (WPPSI, standard score)	11.375	2.550	7.00–17.00	7–13

**Abbreviations**: WPPSI = Wechsler Preschool and Primary Scale of Intelligence, TEA‐CH = test of everyday attention for children.

#### 3.1.2 The Effect of Intervention

**FIGURE 3:**
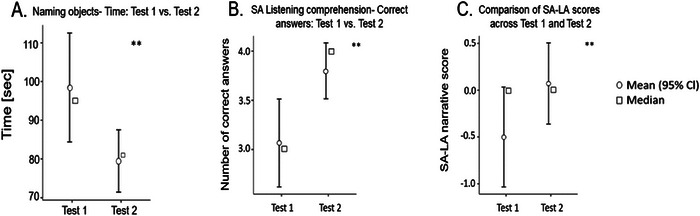
Changes in behavioral scores following DR intervention. A. Decrease in naming objects time following DR; X axis in A. represents the two test time points (Test 1 vs. Test 2) for the time it takes to name the objects, Y axis in A. shows the time in seconds. B. Improved listening comprehension in SA. X axis represents the test time points (Test 1 vs. Test 2), Y axis represents the numbers of correct answers C. smaller gap between SA and LA following DR training. X axis represents the two test time points (Test 1 and Test 2), Y axis represents the score difference (SA narrative comprehension minus LA). Data are shown as mean (95% CI, circles) and median (squares). ** indicates *p* < 0.05.

Paired t‐test results for children participating in the intervention (n = 15) demonstrated that, after Bonferroni correction (*p* < 0.017), significant improvements were observed in SA listening comprehension (t = −3.214, *p* = 0.006) and in the SA–LA difference score (Figure 3C, *p* = 0.01). The decrease in naming objects time (t = 2.169, *p* = 0.048) showed a trend but did not survive correction. See Table 2 and Figure 3.

**TABLE 2:** Paired t‐test results for the effect of DR on EF and language skills.

Ability	Test	Test 1 (A)	Test 2 (B)	T(P)	Contrast
Nonverbal abilities	Matrix (WPPSI, standard score)	10.384 (0.873)	12.230 (0.567)	−1.836 (0.091)	B > A
Language abilities	Vocabulary, LA (WPPSI, raw score)	14.692 (1.654)	16.923 (1.460)	−1.844 (0.090)	B > A
	Vocabulary, SA (WISC, raw score)	18.846 (2.138)	18.846 (1.782)	0.000 (0.019)	B = A
	Narrative comprehension, SA (correct responses)	3.066 (0.228)	3.800 (0.144)	**−3.214 (0.006)**	**B > A**
	Narrative comprehension, LA (correct responses)	3.642 (0.132)	3.714 (0.125)	−0.434 (0.671)	B > A
	Naming objects, time (Loghat Alkeraa, sec)	98.466 (7.191)	79.466 (4.104)	**2.169 (.048**)	A > B
	Naming objects, accuracy (Loghat Alkeraa, number of errors)	0.733 (0.206)	1.133 (0.336)	−1.871 (0.082)	B > A
	Naming letters, time (Loghat Alkeraa, sec)	155.000 (24.753)	128.111 (18.619)	1.185 (0.270)	A > B
	Naming letters, accuracy (Loghat Alkeraa, number of errors)	5.500 (1.711)	6.000 (1.546)	−0.382 (0.714)	B > A
EF	Memory, digit span (CTOPP, standard score)	5.714 (0.690)	5.714 (0.922)	0.000 (1.000)	A = B
	Inhibition, walk/don't walk (correct responses)	2.818 (0.377)	2.818 (0.325)	0.000 (1.000)	A = B
	Inhibition, score (correct responses)	4.071 (0.462)	5.000 (0.419)	−1.410 (0.182)	B > A
	Switching, color (correct responses, raw score)	26.928 (0.780)	27.285 (0.641)	−0.366 (0.720)	B > A
	Switching, animals (correct responses, raw score)	24.928 (1.549)	27.571 (0.768)	−1.851 (.087)	B > A
Supporting EF	Visual attention, sky search (TEA‐CH, age scale)	6.928 (0.766)	7.928 (0.892)	−0.864 (.403)	B > A
	Sky search, time (TEA‐CH, correct per second, age scale)	3.928 (0.683)	5.071 (0.722)	−0.984 (.197)	B > A
	Processing speed, coding (WPPSI, standard score)	10.357 (0.560)	11.928 (.0528)	−1.795 (.096)	B > A

*Note*: Table 2 Significant results are bolded.

### 3.2 Electrophysiological Results

#### 3.2.1 Baseline Measures

**FIGURE 4:**
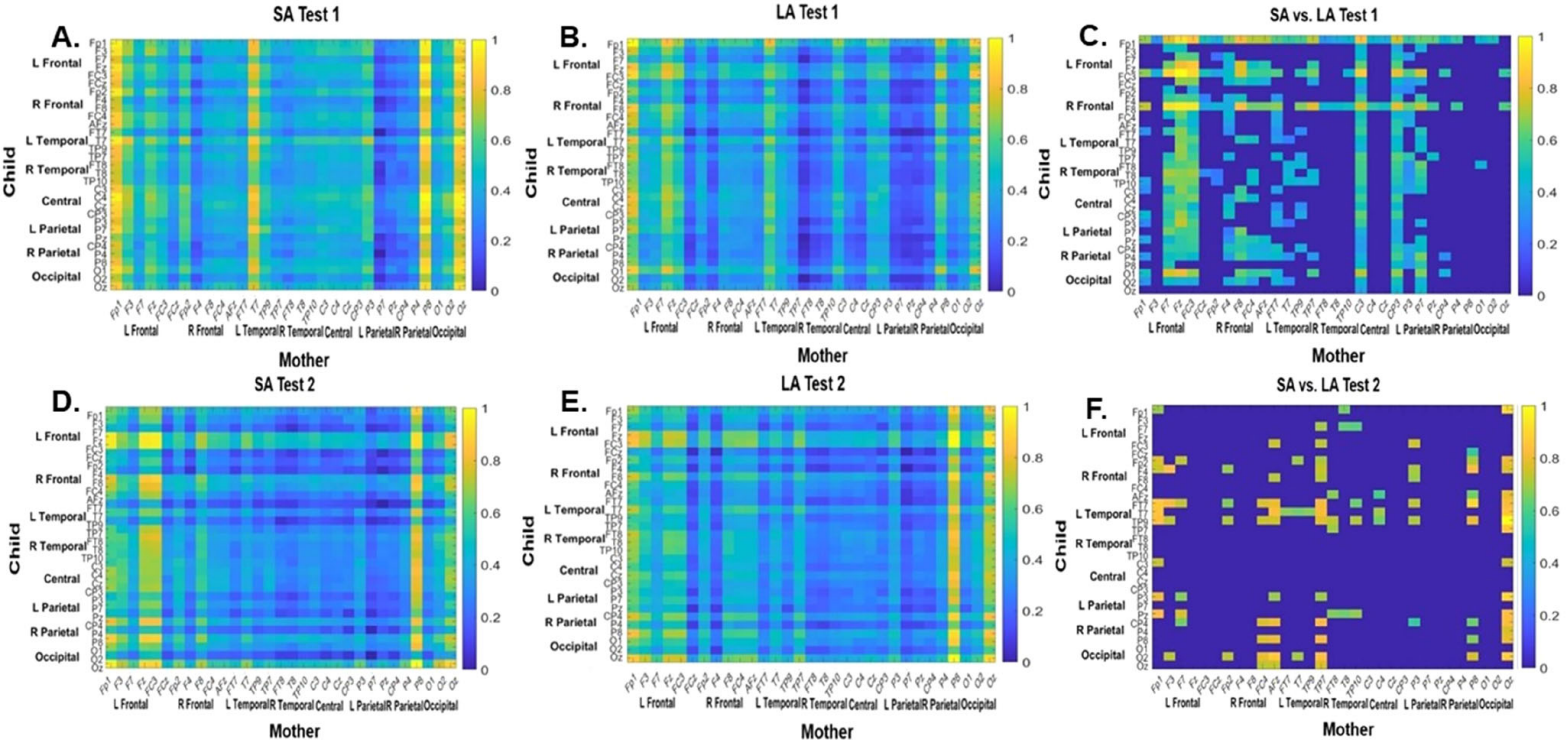
Correlation coefficient matrices for Test 1 and Test 2 during SA versus LA. (A)–(C): correlation coefficient matrices for Test 1 (A. SA, B. LA, C. SA vs. LA) and (D)–(F) for Test 2 (D. SA, E. LA, F. SA vs. LA). The X axis represents the electrodes for the mother and the Y axis represents the electrodes for the child.

Paired t‐test results for the 15 participants performing the intervention pointed to significantly greater correlation coefficient values for the SA compared to the LA matrix. The results indicate a higher mother‐child neural synchronization in frontal and parietal regions (r = 0.807, *p* < 0.001). See Figure 4.

#### 3.2.2 Effect of DR Intervention

Significantly greater correlation coefficient values were found for the SA compared to the LA condition following the DR training. The results indicate that following DR training, brain‐to‐brain synchronization was still greater for SA versus LA (r = 0.814, *p* < 0.001). In addition, a strong correlation was observed between the LA–SA differences in Test 1 and Test 2 (r = .834, *p* < 0.001), indicating a consistency in LA‐SA differences across participants: children who showed larger gaps at Test 1 also tended to show larger gaps at Test 2. See Figure 4.

### 3.3 Correlations Between Changes in Brain‐to‐Brain Synchronization (Brain Regions) and Behavioral Changes Following DR Intervention

**FIGURE 5:**
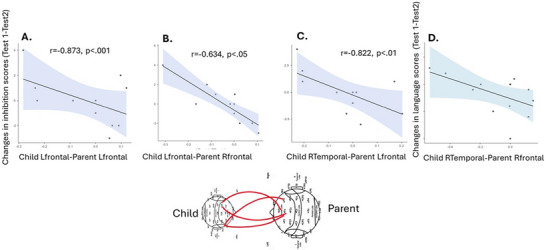
Pearson correlations between changes in child‐parent synchronization in SA versus LA in Test 1 versus Test 2 and changes in behavioral scores following training. (A)–(C): Pearson correlations between changes in synchronization in electrodes in the child and the mother per brain region and changes in inhibition behavioral scores following training; and (D): Pearson correlation with language scores following training. The X axis represents the electrodes for a specific region for a child and his mother, and the Y axis represents the change in behavioral performance scores from pre‐ to post‐intervention.

Differences in brain‐to‐brain synchronization in specific brain regions were defined. Significant changes in synchronization were found in the frontal and temporal brain regions. These regions overlapped with those found significant in the correlation matrix (Figure 4C, F). Synchronization for these brain regions was compared for SA vs. LA in Test 1 and Test 2, where higher values reflect greater changes. Pearson correlations of the difference in synchronization between the conditions in the selected brain regions with the behavioral language and EF changes following intervention revealed significant negative correlations between the left frontal electrodes in the child with the right frontal electrodes in the mother and inhibition scores (Score, r = −0.873, *p* = 0.000, N = 11). Additionally, negative correlations between synchronization between the right temporal electrodes in the child and the left frontal electrodes in the mother, and between the changes in the right temporal electrodes in the child and the right frontal region in the mother, and changes in inhibition scores (Score (r = −0.822, *p* = 0.002, N = 11; r = −0.634, *p* = 0.036, N = 11) respectively, and a trend between the right temporal region of the child and the right frontal region of the mother with the change in language abilities (vocabulary, LA (WPPSI, r = −0.535, *p* = 0.059, N = 13). These results indicate that increased synchronization in these regions was related to better inhibition and language scores in LA following the DR intervention. See Figure 5.

## 4 Discussion

This study aimed to determine the effect of DR in LA on executive functions and language measures in Arabic‐speaking children before they officially acquired reading skills. Using EEG hyperscanning, we assessed the neural synchronization during shared reading sessions in SA and LA before and after the intervention and determined how changes in synchronization related to behavioral performance.

In line with our hypotheses, brain‐to‐brain child‐parent synchronization before training (Test 1) was higher during SA than LA. Following intervention, the gap in synchronization between SA and LA decreased, suggesting that the children became more engaged and cognitively attuned to LA through repeated interactive exposure. The correlation between Test 1 and Test 2 LA–SA differences (r = .834) reflects participant‐level stability, whereas the paired t‐tests (Figure 3C) confirmed a group‐level reduction in the LA–SA difference. Although the median SA–LA difference at Test 1 was close to zero, the mean and confidence intervals revealed variability across children, with some showing a larger disadvantage in LA. Following the intervention, both the mean and median shifted above zero, and the reduction in the LA–SA gap reached significance (Figure 3C), indicating that the intervention reduced variability and improved overall performance in LA. This decreased SA‐LA synchronization following training was also related to improved behavioral scores in EF (inhibition) and language abilities (vocabulary, LA, WPPSI (Wechsler 2002)).

### 4.1 Child‐Parent Brain Synchronization and Executive Function Outcomes

The current findings demonstrate a clear relationship between mother–child brain‐to‐brain synchronization and improvements in EF following DR in LA. Increased synchronization between the child's left frontal region and the mother's right frontal region was significantly associated with better performance on inhibition tasks. These frontal areas are involved in top‐down cognitive control, including goal‐directed attention and self‐regulation (Diamond 2013). The improvement in inhibitory control may reflect the enhanced joint engagement during DR sessions, which promotes shared focus and mutual responsiveness—both critical for the development of EF in early childhood (Howard et al. 2016).

In addition, higher synchronization between the child's right temporal region and the mother's left frontal region was associated with better inhibitory control following DR training (also following [Twait et al. 2019]). These results align with the studies suggesting that interactive language environments support domain‐general cognitive development and language skills (Miyake and Friedman 2012). Dialogic reading, by encouraging children to predict, reflect, and respond during storytelling, may provide an optimal context for practicing cognitive flexibility and inhibitory control (Howard et al. 2016; Flynn 2011; Twait et al. 2019). Studies such as Hutton et al. (2017) and. (Twait et al. 2019) have also shown that shared reading is associated with activation in brain regions responsible for EF, supporting the link between social interaction, attention control, and neural connectivity.

The results of the current study align with the interactive specialization framework (Johnson 2011), which posits that cognitive development is shaped by experience‐driven changes in the coordination between different brain regions. In this case, repeated DR sessions support the emergence of more efficient and synchronized neural processing between the parent and child, especially in regions supporting regulation and control (Gashri et al. 2024). The observed correlations between changes in brain synchrony and behavioral improvements provide converging evidence for the role of socially mediated language experiences in supporting executive functioning in diglossic language contexts.

### 4.2 Child‐Parent Joint Reading and Cognitive Development

In the current study, DR intervention in Arabic led to measurable improvements in children's language and cognitive outcomes. Children demonstrated a trend toward faster naming, though this effect did not survive correction, alongside better performance in an inhibitory control task. These improvements were significantly correlated with increased mother–child brain‐to‐brain synchronization, especially in frontal and temporal regions associated with EF and language processing, respectively. This alignment between neural synchrony and behavioral gains supports the idea that shared reading can promote cognitive development through real‐time interactive engagement (Hutton et al. 2017; Gashri et al. 2024). More specifically, changes in LA versus SA synchronization matrices between the mother's right frontal region and the child's left frontal and temporal areas following training were related to better naming accuracy. In contrast, synchronization between the child's right temporal and the mother's frontal regions was linked to better inhibitory performance. These region‐specific relationships suggest that DR may serve as a facilitator for language and EF practice and reinforcement through social interaction. This dual impact aligns with the interactive specialization framework (Johnson 2011), which emphasizes the role of repeated, meaningful experience in shaping functional brain networks. By promoting joint attention and cognitive coordination, DR in LA appears to provide a supportive environment for developing core academic skills in diglossic learners. These findings are consistent with previous studies showing that DR enhances activation and connectivity in brain regions involved in self‐regulation, memory, and attention control (Howard et al. 2016, Hutton et al. 2017, Gashri et al. 2024).

### 4.3 Study's Limitation

Despite the compelling results, the study has several limitations. First, children in the current study were recruited from a specific region in Israel that spoke a certain Arabic dialect, which may limit the generalizability of the findings. Additional studies replicating the current study design using multiple Arabic dialects are warranted. Second, the study focused only on short‐term changes following the DR intervention. Longitudinal studies are necessary to examine whether these neural synchrony and behavioral outcome improvements are sustained over time. Third, the study did not include a control group of children training on a control training condition or a waiting list group to control for developmental changes, which limits the ability to determine whether the observed changes were specifically due to the DR intervention rather than other external factors. Future studies incorporating waitlist or alternative‐intervention control groups will be important to more conclusively rule out maturation effects and strengthen causal inferences.

Additionally, while the EEG method provides valuable insight into brain‐to‐brain synchrony, it captures only part of the complex dynamic of parent‐child interactions. Combining EEG with other behavioral and observational measures may yield a more comprehensive picture. Finally, the study did not include a competing learning method (e.g., traditional shared reading or other literacy interventions), which would have provided a stronger basis for evaluating whether dialogic reading offers greater benefits compared to alternative approaches. In addition, while the study was sufficiently powered to detect very large correlations, it was underpowered for moderate associations, and these results should therefore be interpreted with caution.

### 4.4 Conclusions

This study demonstrates that DR enhances neural synchrony between mothers and their children, supporting both language and executive function development. While SA elicited higher engagement at baseline, the DR intervention significantly improved synchronization during LA reading, suggesting increased comprehension and joint engagement with more complex linguistic input. These findings highlight the importance of interactive language exposure and suggest that dialogic reading can be a powerful tool for supporting cognitive and linguistic development in diglossic contexts such as Arabic. By fostering mutual engagement and deeper processing through shared storytelling, DR may help bridge the gap between everyday spoken language and the formal language of literacy and schooling.

## Author Contributions

**Georgina Abu Ghanima**: visualization. **Rola Farah**: interpretation. **Tzipi Horowitz‐Kraus**: conceptualization, supervision.

## Supporting information



Supplemental material 1: Examples of spoken Arabic books that parents read with their children from Maktabat El Fanus:
Supplemental material 2: Parents' Guide to Dialogic Reading.
Supplemental material 3: Correlation coefficient matrices for Test 1 and Test 2 during SA versus LA
Supplemental material 4: Correlation coefficient matrices for Test 1 and Test 2 during SA versus LA‐ with different scales for SA: Test1 versus Test2 and LA: Test 1 versus Test 2
