# Supplementary material for: Increased Brain‐to‐Brain Synchronization During Literary Arabic Storytelling Following a Dialogic Reading Intervention: A Hyperscanning‐EEG Study
Source: Brain Behav. 2025 Nov 21;15(11):e71003. doi: 10.1002/brb3.71003 (PMC12638431; doi:10.1002/brb3.71003)

**Supplemental materials**

**Supplemental material 1:** Examples for spoken Arabic books that parents read with their children from Maktabat El Fanus:


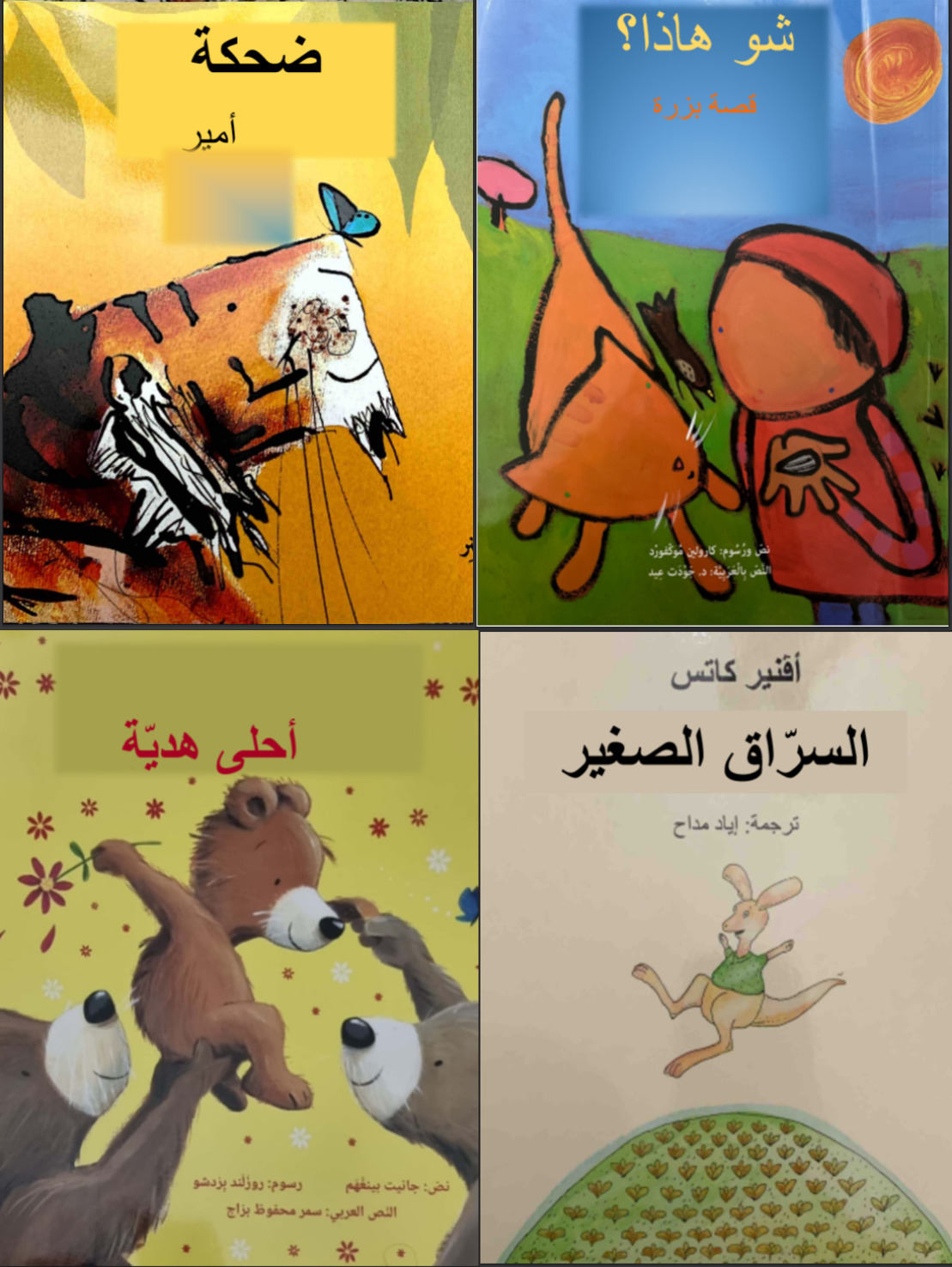


**Supplemental material 2:** Parents' Guide to Dialogic Reading.


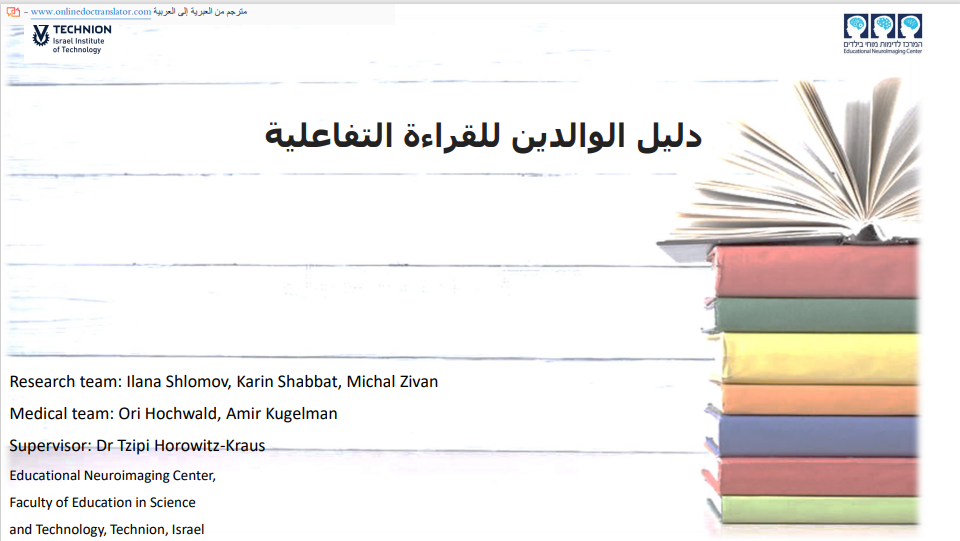


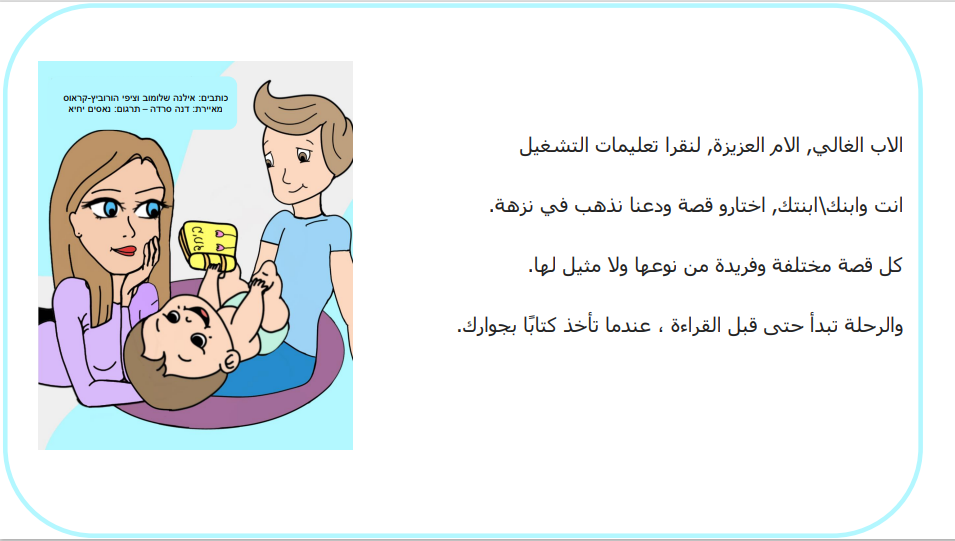


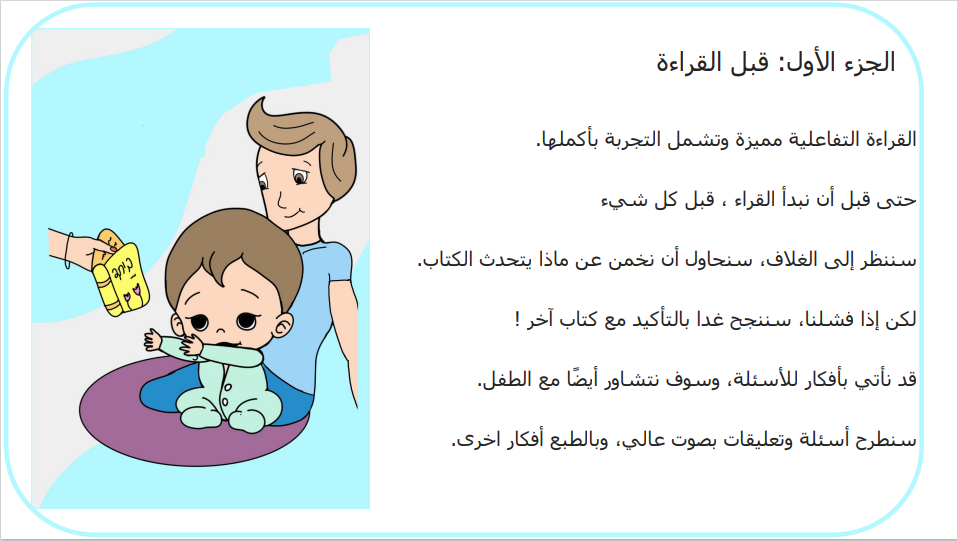


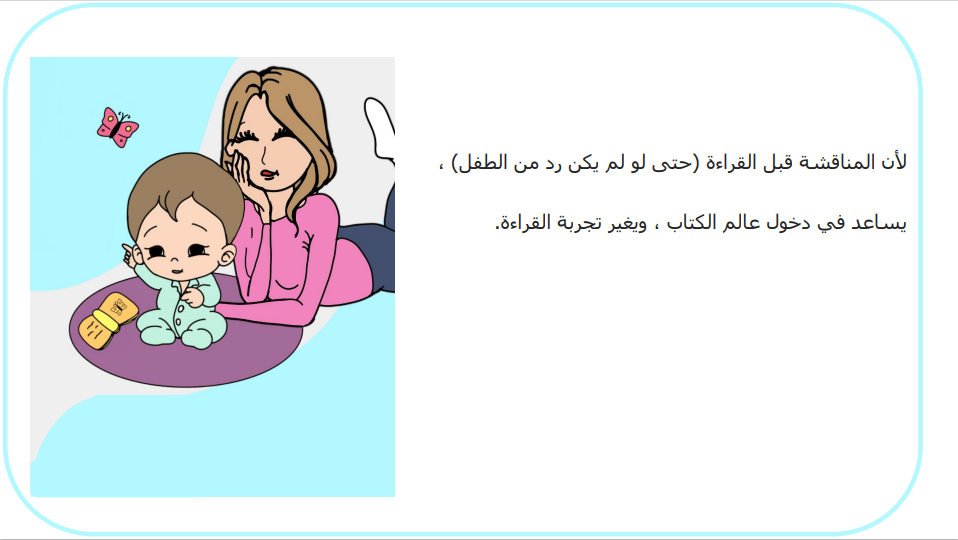


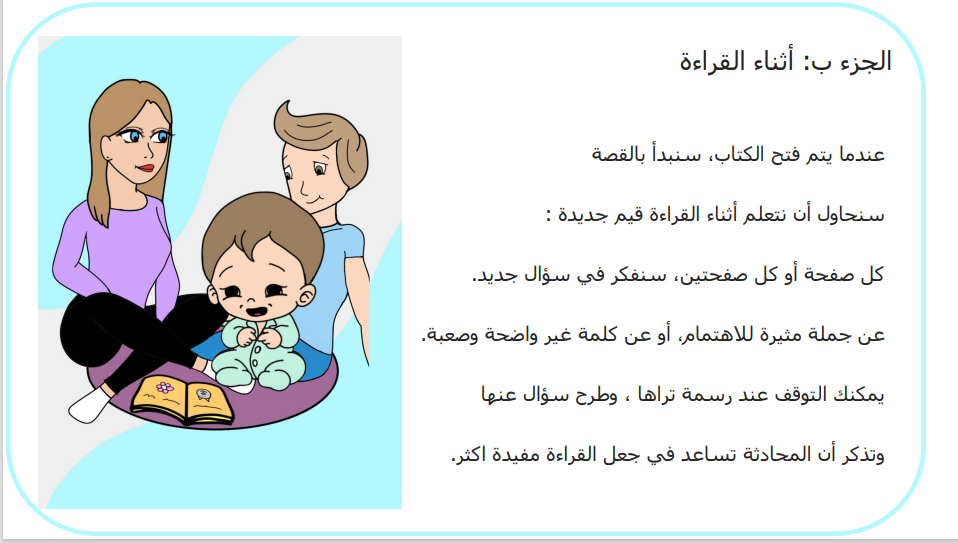


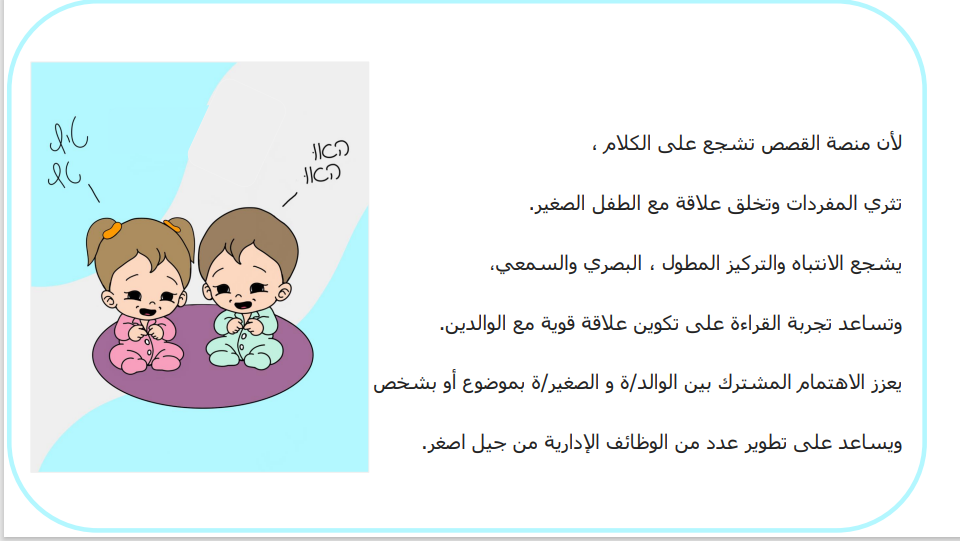


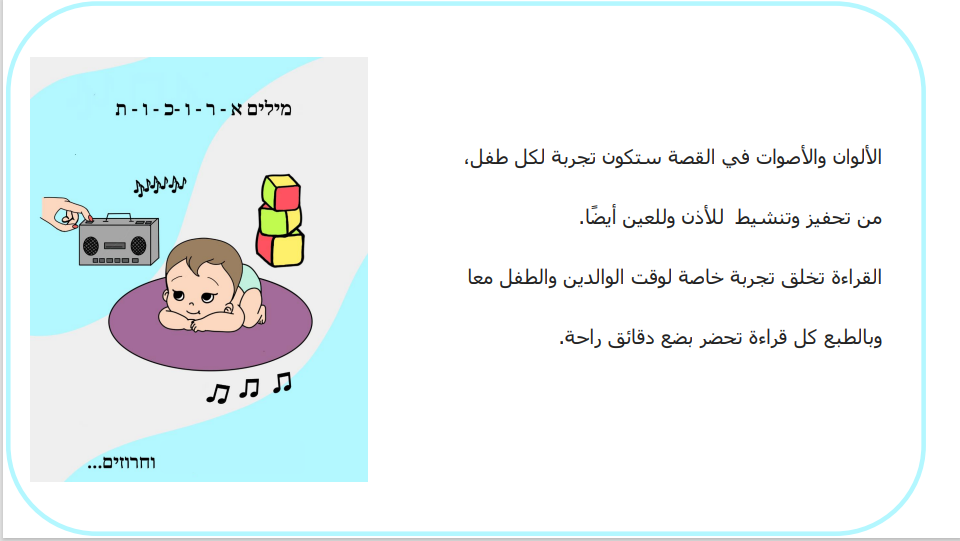


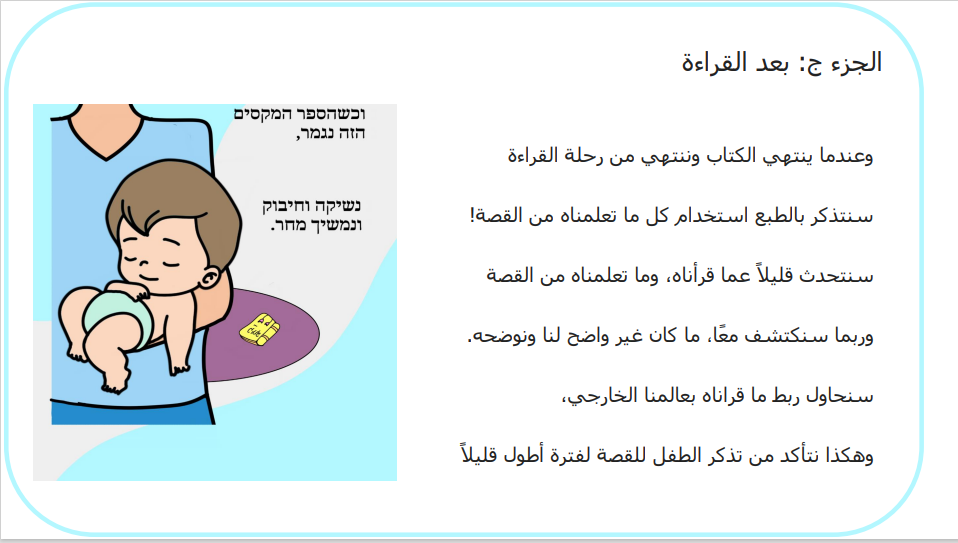


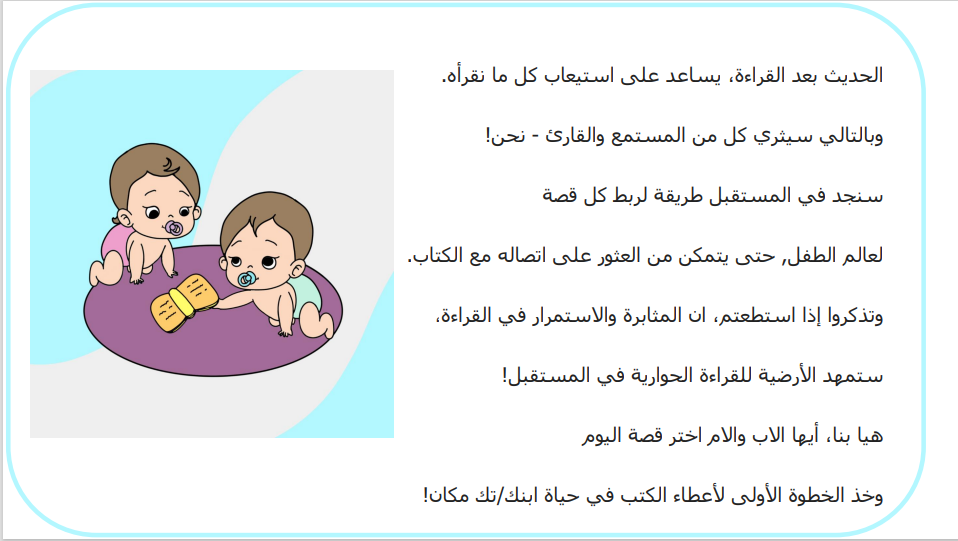


**Supplemental material 3: Correlation coefficient matrices for Test 1 and Test 2 during SA vs LA**

**
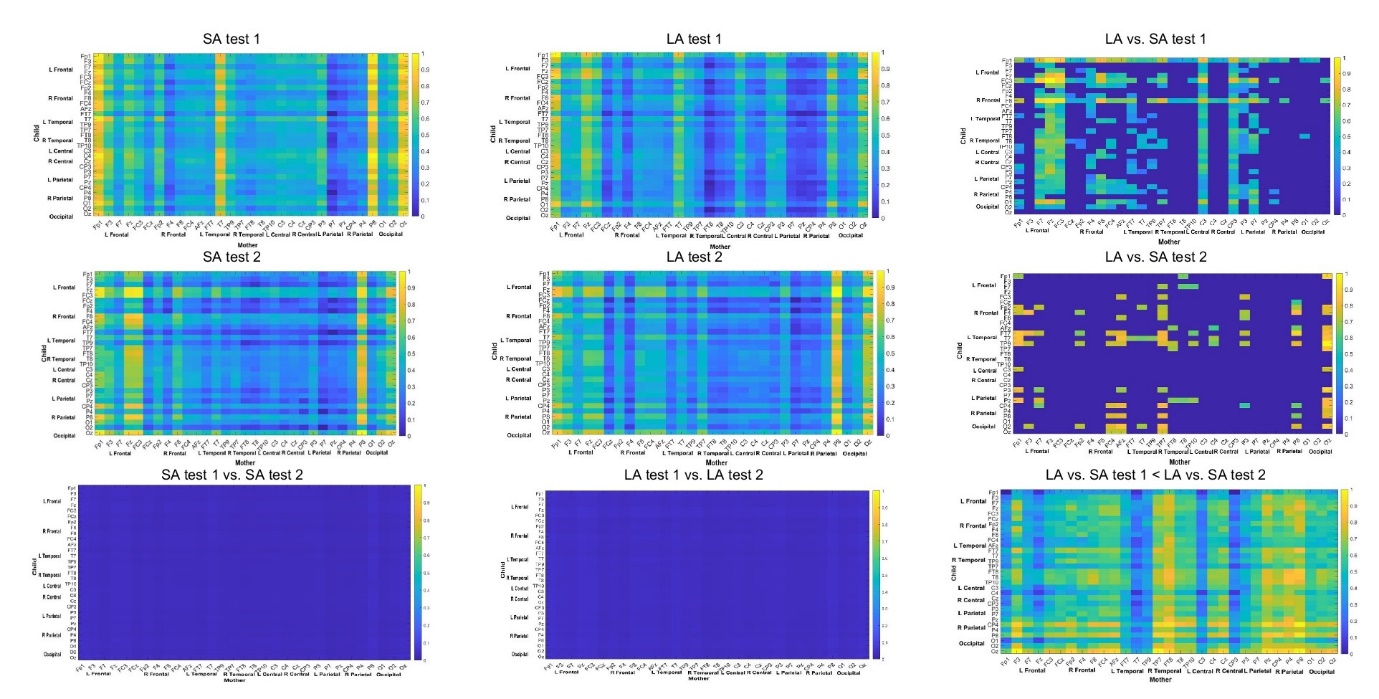
**

**Supplemental material 4: Correlation coeffienct matrices for Test 1 and Test 2 during SA vs LA- with different scales for SA:Test1 vs. Test2 and LA: Test 1 vs. Test 2**


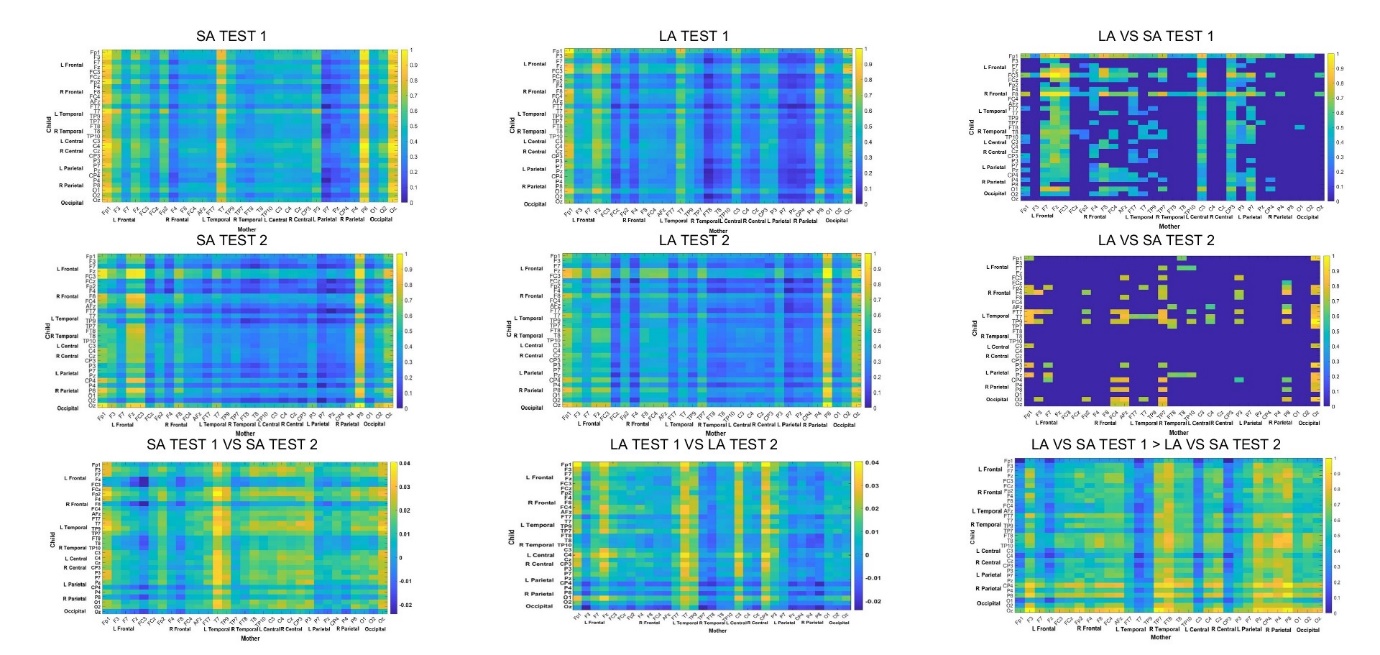

Supplement: Supplementary file 1 — Supplemental material 1: Examples of spoken Arabic books that parents read with their children from Maktabat El Fanus: Supplemental material 2: Parents' Guide to Dialogic Reading. Supplemental material 3: Correlation coefficient matrices for Test 1 and Test 2 during SA versus LA Supplemental material 4: Correlation coefficient matrices for Test 1 and Test 2 during SA versus LA‐ with different scales for SA: Test1 versus Test2 and LA: Test 1 versus Test 2 [file BRB3-15-e71003-s001.docx]
